# Correction to “Effect of Umbilical Cord Mesenchymal Stem Cell‐Derived Mitochondrial Transplantation on Ischemia‐Reperfusion Injury in a Rat Model”

**DOI:** 10.1111/srt.70300

**Published:** 2025-11-29

C. Y. Lee, G. Khan, D. Y. Hyun, S. H. Kim, and E. S. Park, “Effect of Umbilical Cord Mesenchymal Stem Cell‐Derived Mitochondrial Transplantation on Ischemia‐Reperfusion Injury in a Rat Model,” *Skin Research and Technology* 30, no. 9 (2024): e70022, https://doi.org/10.1111/srt.70022.

The ethics statement in the published article was incorrect. The correct statement is presented below:

The animal study protocol was reviewed and approved by the Soonchunhyang University (SCH) Institutional Animal Care and Use Committee (IACUC). Approval Number: SCHBCA2022‐07.

The authors apologize for this error.

